# Machine Learning Analysis of Raman Spectra of MoS_2_

**DOI:** 10.3390/nano10112223

**Published:** 2020-11-09

**Authors:** Yu Mao, Ningning Dong, Lei Wang, Xin Chen, Hongqiang Wang, Zixin Wang, Ivan M. Kislyakov, Jun Wang

**Affiliations:** 1 Laboratory of Micro-Nano Optoelectronic Materials and Devices, Key Laboratory of Materials for High-Power Laser, Shanghai Institute of Optics and Fine Mechanics, Chinese Academy of Sciences, Shanghai 201800, China; yumao@siom.ac.cn (Y.M.); wanglei2016@siom.ac.cn (L.W.); XinChen@siom.ac.cn (X.C.); hqwang@siom.ac.cn (H.W.); zxwang@siom.ac.cn (Z.W.); iv.kis@mail.ru (I.M.K.); 2 Center of Materials Science and Optoelectronics Engineering, University of Chinese Academy of Sciences, Beijing 100049, China; 3 State Key Laboratory of High Field Laser Physics, Shanghai Institute of Optics and Fine Mechanics, Chinese Academy of Sciences, Shanghai 201800, China; 4 CAS Center for Excellence in Ultra-Intense Laser Science (CEULS), Shanghai 201800, China

**Keywords:** 2D materials, machine learning, random forest algorithm, Raman spectrum

## Abstract

Defects introduced during the growth process greatly affect the device performance of two-dimensional (2D) materials. Here we demonstrate the applicability of employing machine-learning-based analysis to distinguish the monolayer continuous film and defect areas of molybdenum disulfide (MoS_2_) using position-dependent information extracted from its Raman spectra. The random forest method can analyze multiple Raman features to identify samples, making up for the problem of not being able to effectively identify by using just one certain variable with high recognition accuracy. Even some dispersed nucleation site defects can be predicted, which would commonly be ignored under an optical microscope because of the lower optical contrast. The successful application for classification and analysis highlights the potential for implementing machine learning to tap the depth of classical methods in 2D materials research.

## 1. Introduction

Transition-metal dichalcogenides (TMDCs) are a class of layered materials analogous to graphene, which have aroused immense interest in the last decade as a promising platform for electronic and optoelectronic applications in the post-Moore era [1,2]. The adjacent layers in these materials are held together by weak van der Waals forces but strong covalent bonding forces inside the layer, making it possible to cleave or synthetize to the limit of a monolayer. Even though TMDCs have been studied for decades in their bulk form, the properties of monolayers and few-layers with ultrathin thickness differ dramatically from the macroscopic material characteristics, being the biggest reason for renewed interest in this material class. Compared to their bulk counterpart, monolayers of many TMDCs (such as MoS_2_, WS_2_, WSe_2_ and MoSe_2_) are especially exciting since they are direct band gap semiconductors, making them ideal candidates to replace silicon for device applications, such as light-emitting diodes, photodetectors and photodiodes [3]. The chemical vapor deposition (CVD) method provides a convenient and controllable way to grow high-quality and large area 2D materials at a reasonable cost, which has been earmarked as the process that will deliver scalable production [4,5]. Nowadays, continuous films of 2D materials compatible with current silicon-based microfabrication processes are greatly needed for industrial electronic and optoelectronics applications [6,7,8]. However, the present uniformity of as-grown monolayer films is still inadequate, such as structural differences and poor controllable layer distribution, etc. It is a remarkable fact that the introduced cracks that appear in synthesis processes can adversely affect the device performance and directly increase the chip failure risk [9]. These factors greatly limit the further applications of these materials. As a consequence, it is essential to check the uniformity of the obtained 2D materials. It is necessary to locate and distinguish the monolayer continuous films and the random crack areas, as well as bilayer areas prone to be introduced in the growth process, in order to better understand and improve the growth process [10].

To identify these areas with thickness differences, the most typical methods are atomic force microscopy (AFM) [11,12], optical microscopy (OM) [13,14,15], differential reflectance spectra [16,17] and Raman spectra [18,19]. AFM is a versatile method used to measure the thickness of 2D materials; however, the materials are easily destroyed due to the sliding of the tip on the sample surface when working on contact mode, while the tapping mode takes a relatively long time to measure even a small area [20,21]. Time-domain terahertz spectroscopy is an emerging technique for imaging 2D materials, but the comparatively large spot size hinders the identification of laterally small flakes [22]. In such a situation, an accurate, versatile and nondestructive method is highly desirable not only in fundamental research but also for practical applications. Recently, machine learning approaches have attracted considerable attention for solving various problems in materials science and optical engineering. OM using red–green–blue (RGB)-based optical contrast combined with machine learning has shown emerging potential in identifying and determining the thicknesses of 2D materials. Lin et al. first used the support vector machine (SVM) method to learn the contrast information of optical images to determine the layer numbers of graphene and MoS_2_ [23]. Later, clustering analysis [24] and the convolutional neural network (CNN) [25,26,27] also joined in this stage play, expanding the identification types and application scenarios of 2D materials. However, because the accuracy of using optical images to determine the layer number based on machine learning is not too high, the previous researchers mainly regarded it as an initial screening to reduce manual work [25]. The attempt to use photoluminescence (PL) imaging and computer vision techniques to analyze monolayer TMDCs also enlightened us to combine the research methods of intrinsic material properties with new technologies to obtain information about molecular structure and layer number simultaneously [28]. Nowadays, based on the relation between the Raman frequency shifts of the $E_{2g}^{1}$ and A_1g_ peaks (TMDCs) and those of the 2D and G peaks (graphene), Raman spectroscopic mapping has been widely used to identify the thickness and confirm the uniformity of 2D materials [29,30,31,32]. As a high-resolution imaging technique, it does give us more information to study the nature of matter. However, it is difficult to identify these defects in the selected area and at the same time simply using one-side Raman frequency shifts. Hence, we consider solving this problem by introducing a machine learning approach to use more Raman features for the simultaneous identification.

To our knowledge, we are the first to present a recognition method to distinguish the monolayer continuous film and random defect areas of 2D semiconductors using the machine learning method with Raman signals. Compared to other unsupervised techniques, the supervised machine learning represented by random forest can not only reduce the computational expense and time but also achieves high accuracy. In the introduction process for the random forest algorithm, we use several Raman characteristics extracted from spatial mapping results as the input variables and the sample thickness type as the output variable for generating the decision trees. The successful application of a machine learning approach to the classification and analysis of the CVD-prepared MoS_2_ highlights the potential of this method for 2D materials research.

## 2. Materials and Methods

### 2.1. Synthesis of Monolayer MoS_2_ Continuous Film

The monolayer MoS_2_ continuous film mentioned in this paper was synthesized via the CVD method similar to our previous work [33]. The film was grown using MoO_3_ powders (99.97% Sigma Aldrich, St. Louis, MO, USA) as the molybdenum source and sulfur powders (99.98%, Sigma-Aldrich, St. Louis, MO, USA). First, MoO_3_ and sulfur powders were loaded into two separate Al_2_O_3_ crucibles, which were located at the center and the upstream of a dual-temperature-zone tube furnace with a diameter of 100 mm. A piece of Si substrate with thermally grown 300-nm-thick SiO_2_ was loaded at the downstream. Before the film growing, the quartz tube was evacuated to 4000 Pa at room temperature. Then, the temperature of the MoO_3_ was increased to 670 °C and the temperature of the sulfur was increased to 190 °C with 50 sccm of argon gas, and maintained for 10 min. Finally, the furnace was naturally cooled down to room temperature.

### 2.2. Characterization and Measurements

We carried out PL, AFM and Raman measurements. The PL signals were collected by a confocal microscopy setup (LabRAM HR Evolution, Horiba Co., Kyoto, Japan) with a 532 nm continuous-wave (CW) laser of a frequency-doubled Nd:YAG laser. The height profiles were measured using AFM taken by an FM-Nanoview6800 (FSM-Precision Co., Suzhou, China) in tapping m ode. Raman spectra were obtained using the same confocal microscopy system equipped with a programmable scanning stage with a 532 nm CW laser (Changchun New Industries Optoelectronics Tech. Co., Changchun, China) as the excitation source. We chose the 100× objective lens (MPLFLN 100×, NA = 0.9) and set the laser power below 1 mW to avoid local heating and undesirable oxidation of the sample. The scanning step was set as 0.2 μm and the integration time was also carefully optimized to obtain an adequate spectrum resolution and a satisfactory signal-to-noise ratio, while maintaining acceptable data acquisition duration and avoiding drift.

## 3. Results and Discussion

**Figure 1:**
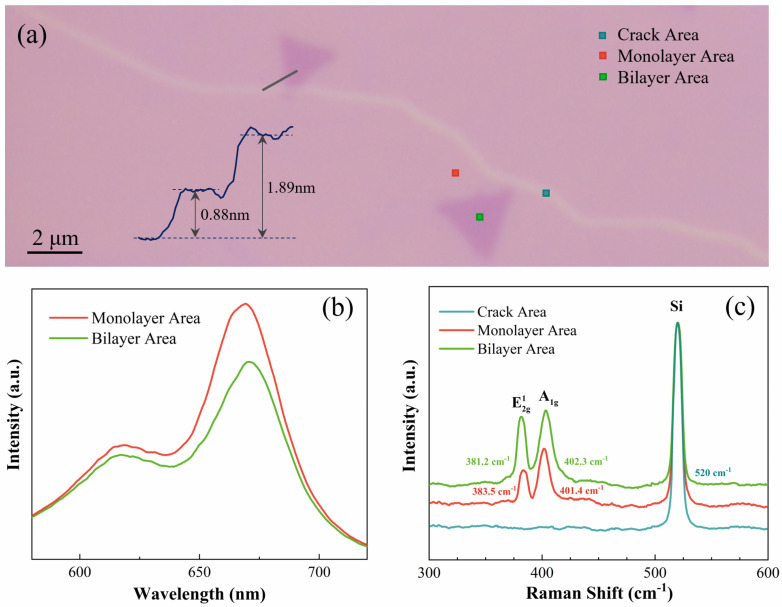
(**a**) Optical image of the MoS_2_ sample. The inset shows the height profile, and the atomic force microscopy (AFM) profile is taken along the gray line drawn on the optical image. (**b**) Photoluminescence (PL) spectra of the monolayer and bilayer areas. (**c**) Raman spectra of the monolayer, crack and bilayer areas.

As shown in Figure 1a, the MoS_2_ monolayer continuous film has a relatively smooth surface. The thickness of the monolayer region is around 0.88 nm as confirmed by AFM, which corresponds to an interlayer S–Mo–S layer [19]. The heights of the undertint line and dark triangle areas were found to be crack and bilayer defects, respectively. The PL spectra in Figure 1b show two peaks at 670 nm and 620 nm corresponding to A (1.9 eV) and B (2.0 eV) direct excitonic transitions with the energy split from the valence-band spin–orbital coupling, respectively. The bilayer shows a decline in PL intensity compared with the monolayer [34]. As shown in Figure 1c, two Raman-active modes, $E_{2g}^{1}$ and A_1g_, exhibit significant differences, and the Raman frequency difference of the monolayer sample is ~17.9 cm^−1^, while that of the bilayer sample is ~21.1 cm^−1^. This is consistent with previous results for mechanical exfoliation (ME)-prepared and CVD-prepared samples, implying this difference is universal between samples obtained from different preparation methods [35,36,37]. The cracks nucleated at sulfur vacancies propagate along the energy-favored zigzag directions upon the relatively fast temperature-drop-induced thermal strain, which results in an orientation-specific fracture behavior [38]. In addition, the appearance of an $E_{2g}^{1}$ mode proves that all the monolayer and bilayer samples are 2H-MoS_2_ [39]. In the completely exposed internal area of the crack, the Raman signal of the Si substrate is mainly collected. The peak around 520 cm^−1^ is attributed to the Si mode. Similar to the case of multilayer graphene, the Raman signals from the Si substrate can be absorbed by the MoS_2_ flakes, which makes the intensity of the Si mode monotonously decrease from the bare substrate to the monolayer and bilayer MoS_2_ flakes [40,41]. In Figure 1c, the intensity of the Si mode is normalized to display the spectral information more intuitively like the previous work [42].

**Figure 2:**
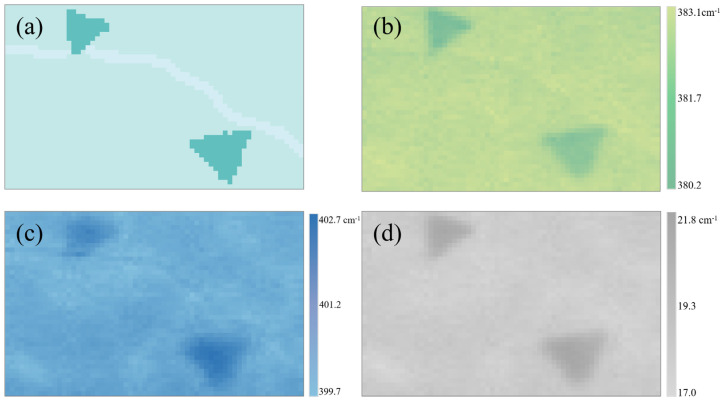
(**a**) K-means algorithm clustered image for the selected sample with monolayer (cyan), bilayer (dark cyan), and crack (light cyan) regions. Raman spectral mapping of the (**b**) Pos($E_{2g}^{1}$) and the (**c**) Pos(A_1g_). (**d**) Raman spectral mapping of the frequency difference between the Pos($E_{2g}^{1}$ ) and the Pos(A_1g_).

The pixels of the optical image in Figure 1a were reduced to be consistent with the collection points of the spectra by bicubic sharper process. Then, the reserved pixels could be clustered using the k-means algorithm [43], which can partition all pixels into three clusters with each cluster having a mean value, and pixels in one cluster are closest to the corresponding mean value among the cluster means [44]. Combined with the AFM heights, three regions with crack, monolayer and triangle bilayers can be identified as shown in Figure 2a. When using k-means clustering to classify different regions of the optical image, in addition to ensuring sufficient optical contrast, the clustering also depends on the AFM data to ensure effective classification. Nowadays, spectroscopy is the backbone of research in such diverse fields, ranging from physics to engineering, chemistry and biology [45]. Compared to relying on AFM to determine thickness, the more convenient way is to use the potentiality of classical spectroscopy research methods to obtain information about molecular structure, chemical composition and even layer number simultaneously.

In the research methods for 2D material properties, Raman spectroscopy is the most commonly used technique in many fields, since it allows the essential characteristics of matter that are invisible by standard OM and AFM to be viewed [46]. Ultralow-frequency Raman spectroscopy has been used to reliably determine layer numbers of TMDC flakes, but this technique requires expensive adapters and nonstandard equipment setup [47]. Therefore, it is of vital importance to look for a suitable technique using the standard Raman system. Generally, high-frequency Raman peaks of lattice vibrations (i.e., phonons) in TMDCs exhibit several prominent features, including frequency, intensity and full width at half maximum, which contain useful information in characterizing the physical and chemical properties of the materials. Figure 2b,c show the Raman spectral mapping of the $E_{2g}^{1}$ and the A_1g_ peaks. From these two mappings, we can find the differences between the monolayer and bilayer areas. The frequency of the $E_{2g}^{1}$ peak decreases, while that of the A_1g_ peak increases with increasing layer number. The observed blueshift of the A_1g_ peak originates from the constraint of atom vibration by the interlayer van der Waals force in MoS_2_, whereas the stacking-induced structure changes or long-range Coulomb interlayer interactions account for the redshift of the $E_{2g}^{1}$ peak [18]. Therefore, as shown in Figure 2d, the Raman frequency difference between the $E_{2g}^{1}$ and A_1g_ peaks can be used as a fingerprint feature to identify the monolayer and bilayer MoS_2_ regions of the flakes [19]. However, the crack area cannot be identified straightforwardly by only using the frequency shifts of the $E_{2g}^{1}$ or A_1g_ peaks.

**Figure 3:**
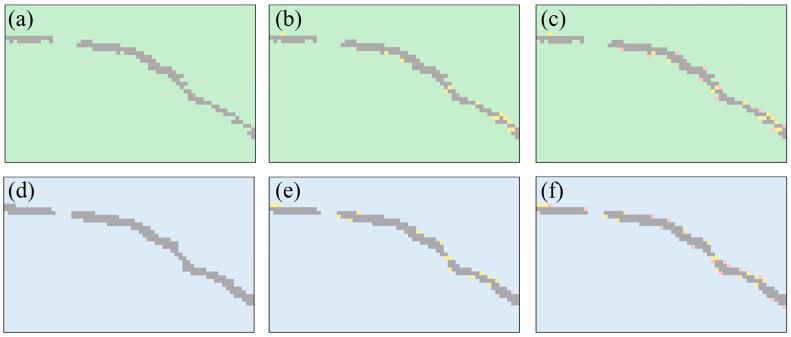
Raman spectral intensity mapping of the $E_{2g}^{1}$ and the A_1g_ peaks. Gray areas and the newly added yellow and red areas indicate the situation when the $E_{2g}^{1}$ and the A_1g_ peak intensity drops below (**a**,**d**) 68%, (**b**,**e**) 70% and (**c**,**f**) 72% of the monolayer signal, respectively.

The great discrepancies in Raman intensity from crack to monolayer inspired us to consider more features extracted from Raman spectra to realize the simultaneous identification of these three regions. It is worth noting that when the detection position controlled by the scanning stage moves to the boundary of two regions, i.e., the junction of the crack and monolayer, it will inevitably collect signals both from the crack and monolayer MoS_2_ at the same time, which makes it difficult to classify different areas based on Raman intensity by setting the threshold manually. We chose the average value of the $E_{2g}^{1}$ peak intensity of a large monolayer MoS_2_ area as the reference intensity, and all thresholds (a fixed percentage) were set based on this value. The Raman spectral intensity mappings of the $E_{2g}^{1}$ peak are shown in Figure 3a–c and the thresholds are 68, 70 and 72%, respectively. It is not difficult to see that when the threshold changed slightly, the predicted information of the crack area and the monolayer area also changed. This means that an effective evaluation tool is lacked for judging the rationality of the artificially set thresholds. In addition, the monolayer sample generally has two Raman peaks, $E_{2g}^{1}$ and A_1g_, under the excitation of green or blue lasers with proper power. When using the same intensity threshold of the A_1g_ peak to make judgments, there are also some differences in the conclusions drawn compared to the $E_{2g}^{1}$ peak, as shown in Figure 3d–f, which further increase the difficulty of manual processing. The rising machine learning approaches can help to successfully extract and analyze the multiple Raman characteristics among many samples to address this problem.

**Figure 4:**
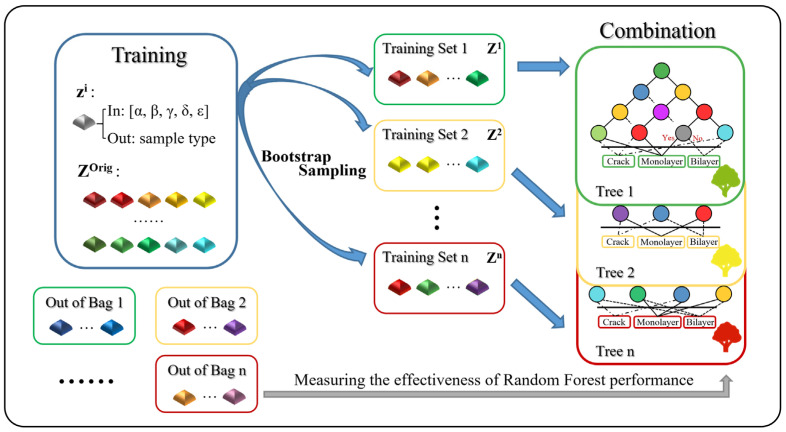
Basic architecture of the learning procedure in the random forest method. Each small square represents a spatial measurement point carrying Raman characteristic information. The subtraining sets from 1 to n are acquired by a bootstrap sampling process, and then decision trees based on these subtraining sets can be built. The out-of-bag data of each tree can be used to estimate the effectiveness of the trained random forest model.

The introduction of machine learning enables computers to tackle problems involving knowledge of the real world and make decisions that appear correct. Here we implement the random forest algorithm (Figure 4) to search for a hidden correlation that may exist between the sample types and the characteristic data obtained from the spatial Raman mapping. This method has been successfully applied on a PL spectra study [48]. Compared to other classification procedures, the random forest machine learning approach has the advantage of high classification accuracy [49]. Furthermore, it can determine variable importance and model complex interactions among predictor variables [50]. Here, we define *z^i^*, including five types of spectral information (*α*, *β*, *γ*, *δ* and *ε*) as the input variables: *α* and *β* are the intensity and frequency of the $E_{2g}^{1}$ peak, respectively; *γ* and *δ* are the intensity and frequency of the A_1g_ peak, respectively; *ε* is the Raman frequency difference between the two peaks previously mentioned. The sample types of crack, monolayer and bilayer are defined as output variables, which are acquired from the k-means algorithm results. The *Z^Orig^* = [*z^1^*, *z^2^*, *z^3^*, …, *z^i^*, …, *z^k^*] are all training data for machine learning. Bootstrap sampling is used to expand a moderate number of data sets into a large volume of data sets required to improve the classification accuracy, which is a resampling technique used to estimate statistics on a population by sampling a dataset with replacement. Concretely speaking, we create n decision trees by generating new data sets *Z^1^*, *Z^2^*, ..., *Z^n^*, using n data sets randomly extracted from the original *Z^Orig^* with duplication permitted. For example, *Z^1^* = [*z^3^*, *z^7^*, *z^16^*, *z^g^*, ..., *z^i^*] and *Z^2^* = [*z^5^*, *z^5^*, *z^15^*, ..., *z^k^*], where *g* and *i* are integers (≤*k*), and some data are allowed to appear multiple times (e.g., *z^5^* appears twice in *Z^2^*). As a result, bootstrap sampling can maintain the original distribution of the data and make the generated training sets independent of each other, thereby significantly improving accuracy [51].

Random forest is an ensemble learning algorithm that uses a group of decision trees built by the subtraining sets as weak individual learners of randomly sampled training data. Each decision tree has multiple nodes, and the threshold values of the variables at each node are computationally determined to yield the largest information gain. Generally speaking, the greater the information gain, the greater the “purity improvement” obtained using this feature variable. Since one individual decision tree typically exhibits high variance and tends to overfit, random forest can achieve reduced variance by combining diverse trees, hence yielding a better model overall. Moreover, the out-of-bag data that are not used in the training process for each decision tree can be used to estimate the skill and effectiveness of the trained random forest model. The whole algorithm is conducted in Python using the open-source pandas and scikit-learn machine learning libraries [52].

**Figure 5:**
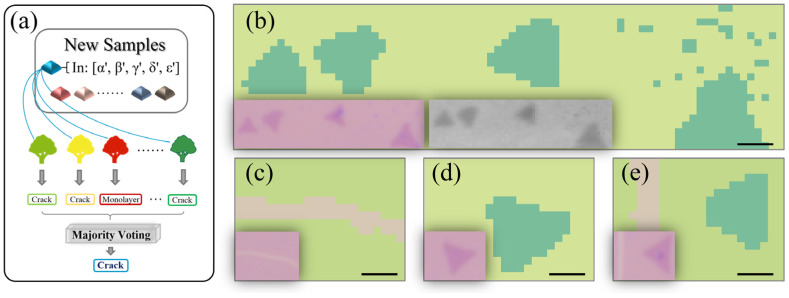
(**a**) Basic architecture of the prediction procedure in the random forest method. The new samples from the untrained data are judged through each tree one by one, and the final output results are acquired by the majority voting process. (**b**–**e**) The predicted pictures for different samples with crack (grown), monolayer (grass green) and bilayer (dark green) areas. The dispersed dots shown in Figure 5b are predicted to be bilayer. The inset figures in Figure 5b show the corresponding optical micrograph (left inset) and Raman mapping of the input variable *ε* (right inset). The other inset figures show the corresponding optical micrographs. Scale bars indicate 1 μm.

Figure 5a shows the prediction procedure for the random forest method. After generating the whole forest, new samples from different positions carrying the input Raman characteristic information will be judged through the formed decision trees one by one. Since the training sets are different from each other, they may give different judgments for one sample by different trees. The final output result is generated by the democratic majority voting which can acquire a dramatically reduced variance. After the optimal parameter combination is fixed, it can immediately give a classification result for the new input data. The accurate measurement of Raman spectra and the use of a sufficient number of training sets enable the constructed random forest to obtain a relatively high accuracy rate. As shown in Figure 5b–e, we choose different sample areas on several pieces of substrate to test our model, and the classification results via the random forest method are successful in distinguishing the monolayer, crack and bilayer areas. In the central areas of these three regions, random forest can provide reliable results. However, due to the relatively small number of detection points at the boundary regions, the result accuracy is not particularly high. Compared with the Raman mapping of the input variable *ε* in Figure 5b, we find that the proposed model successfully retains the accuracy of Raman shifts to effectively identify monolayer and bilayer areas. Benefitting from the high-speed computing power of the computer, the random forest algorithm can be easily and continuously strengthened by increasing the amount of data. As the number of learning data of insufficient sample types are increased, the recognition accuracy is expected to be further improved [48].

Furthermore, some dispersed dots in Figure 5b are easily predicted, which would be commonly ignored under optical microscope because of the lower optical contrast. As reported in the previous work [53], several stages were observed during the MoS_2_ atomic layer growth. Initially, some small domains were nucleated at random locations on the substrate. Then, the nucleation sites continued to grow and formed boundaries when two or more domains met, resulting in partially continuous films. The as-grown films are predominantly monolayer, with small areas consisting of two or more layers at the preferred nucleation sites [42,53], which explains the manifested bilayer Raman characteristics of these areas located on the monolayer continuous film. This suggests that prediction via the random forest possesses the application potential to view subtle differences through the material’s basic features. By using a shorter wavelength laser and a larger numerical aperture objective, it is expected that the spatial resolution will be further improved, which makes it possible to identify defects that are difficult to find and/or determine only by optical images. A smaller laser spot is ideal for analyzing the microscopic characteristics of the sample. In theory, the higher the spatial resolution, the more precise the micro-area spectral information that can be obtained. The random forest algorithm is not a conservative and fixed tool and it can be flexibly adjusted according to our actual needs to adapt to different scenarios. During the Raman spectra acquisition process, it is better to ensure the stability of the experimental conditions, so as to ensure the accuracy to the greatest extent.

To further demonstrate the performance of our built random forest model, the receiver operating characteristic (ROC) and precision-recall (PR) curves are analyzed for crack/others and bilayer/others, respectively [25]. We use pre-labeled new data that does not appear as the testing set to calculate the accuracy, which is used to evaluate the performance of the trained model. The ROC curve is a popular method for accuracy assessment because it is comprehensive, understandable and visually attractive [54,55]. This method uses the area under the curve (AUC) for quantitative assessment, which plots 1-specificity on the x-axis against sensitivity on the y-axis. The range of the AUC varies from 0.5 to 1.0 and a perfect predictor gives an AUC score of 1, while a predictor that makes random guesses like coin tossing has an AUC score of 0.5. While the PR curve shows the trade-off between precision and recall for different thresholds. A system with high recall but low precision will return many results, but most of its predicted labels are incorrect in comparison to the training labels. However, a system with high precision but low recall is just the opposite, returning very few results, but most of the predicted labels are correct in comparison to the training labels. An ideal system with high precision and high recall will return many results, with all results labeled correctly.

**Figure 6:**
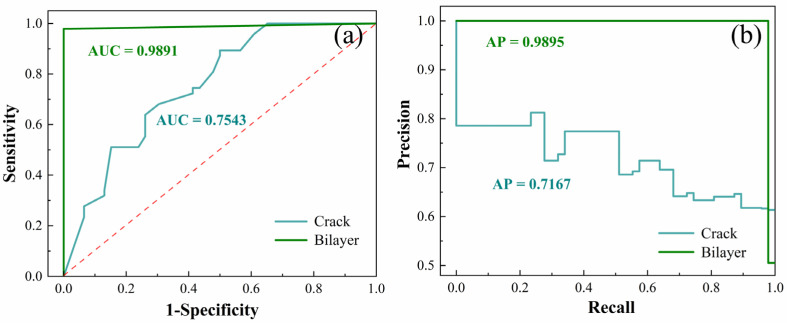
(**a**) Receiver operating characteristic (ROC) curves for the crack and bilayer identification only use the characteristic of the Raman frequency difference. Cyan and green curves show the ROC for two-class classifications of crack/others and bilayer/others, respectively. The red line corresponds to the situation of a random guess. (**b**) Precision-recall (PR) curves for the crack and bilayer identification only use the characteristic of the Raman frequency difference.

As shown in Figure 6a, when using only the Raman frequency difference between the $E_{2g}^{1}$ and A_1g_ peaks, the AUC is 0.9891 for bilayer/others but is only 0.7543 for crack/others, which means that the Raman frequency difference can recognize bilayer samples extremely well but is not good for the cracks. Uniformly, the PR curves in Figure 6b also prove this point, in which the average precision (AP) values are 0.9895 and 0.7167 for bilayer/others and crack/others, respectively. However, when taking all the input variables (*α*, *β*, *γ*, *δ* and *ε*) into consideration, the numerical values of AUC and AP are 0.9852 and 0.9867 for crack, and 0.9902 and 0.9914 for bilayer, which means that this technique based on the random forest method can successfully characterize and confirm the monolayer, crack and bilayer areas at the same time.

Generally, unsupervised techniques, such as CNN and U-Net, are more robust, but they require immense training data and higher computing resources. However, supervised machine learning, like SVM and random forest, deals with prelabeled training data, which not only reduces the computational expense and time but also achieves high accuracy especially when the dimension of the feature vector is not very large [56]. Compared with using the optical contrast via pixel intensities of red, green and blue, the Raman features can directly describe and reflect the intrinsic characteristic differences in materials, which means that even a reduced number of training sets can also help us to get relatively accurate results in a short time. Among the machine learning algorithms, the random forest algorithm has been proven to have unique potential in processing spectral data, with benefits due to its high accuracy and strong resistance to over-fitting. The introduction of machine learning makes it possible to continuously learn in spectroscopy research.

This work is a preliminary step and an attempt to combine machine learning methods with traditional Raman spectroscopy, but it has great migration possibilities for other 2D materials and even bulk materials. Since the Raman spectra of various defects in imperfect 2D materials exhibit different changes, we expect that this method will play a greater role in the characterization of more complex material property control engineering, such as doping, oxidation, mechanical deformations, etc. Machine learning algorithms can be used to build databases under different equipment and experimental conditions, which can better help us analyze and compare experimental data. At the same time, the abundant information in the spectra makes it possible for machine learning to solve different problems by extracting different multi-dimensional variables. The power of machine learning is rapidly transforming modern science, and we can anticipate more exciting results stemming from this interplay between machine learning and the physical sciences [57]. Nowadays, the confluence of many traditional and emerging disciplines, for example, nano-manufacturing, big data technology, computer science and artificial intelligence, is expected to lead the trend in the theoretical and experimental advances in exploring 2D materials, and usher in abundant research opportunities for developing novel 2D devices and systems.

## 4. Conclusions

In this study, we demonstrated an effective method based on a random forest algorithm to classify monolayer MoS_2_ continuous film, random crack and bilayer areas from the variables extracted from Raman spectra. The random forest method was used to analyze multiple Raman features to identify samples, solving the problem of ineffective identification of samples by just one specific variable. It can successfully determine the importance of a certain characteristic variable and some dispersed defect dots can also be predicted, which would commonly be ignored under an optical microscope. By taking peak intensity and frequency information into consideration at the same time, a high accuracy rate is obtained. The method developed in this work can also be used for other 2D materials and can provide a valuable reference for material characterization in several fields.
